# Clinical impact of implementing a rapid-response team based on the Modified Early Warning Score in wards that offer emergency department support

**DOI:** 10.1371/journal.pone.0259577

**Published:** 2021-11-11

**Authors:** Lorena Micheline Alves Silva, Diego Marques Moroço, José Paulo Pintya, Carlos Henrique Miranda

**Affiliations:** Division of Emergency Medicine, Department of Internal Medicine, Ribeirão Preto School of Medicine, São Paulo University, Ribeirão Preto, SP, Brazil; St. Michael’s Hospital, CANADA

## Abstract

**Background:**

Emergency department (ED) crowding is a frequent situation. To decrease this overload, patients without a life-threating condition are transferred to wards that offer ED support. This study aimed to evaluate if implementing a rapid response team (RRT) triggered by the modified early warning score (MEWS) in high-risk wards offering ED support is associated with decreased in-hospital mortality rate.

**Methods:**

A before-and-after cross-sectional study compared in-hospital mortality rates before and after implementation of an RRT triggered by the MEWS ≥4 in two wards of a tertiary hospital that offer ED support.

**Results:**

We included 6863 patients hospitalized in these wards before RRT implementation from July 2015 through June 2017 and 6944 patients hospitalized in these same wards after RRT implementation from July 2018 through June 2020. We observed a statistically significant decrease in the in-hospital mortality rate after intervention, 449 deaths/6944 hospitalizations [6.47% (95% confidence interval (CI) 5.91%– 7.07%)] compared to 534 deaths/6863 hospitalizations [7.78% (95% CI 7.17–8.44)] before intervention; with an absolute risk reduction of -1.31% (95% CI -2.20 –-0.50).

**Conclusion:**

RRT trigged by the MEWS≥4 in high-risk wards that offer ED support was found to be associated with a decreased in-hospital mortality rate. A further cluster-randomized trial should evaluate the impact of this intervention in this setting.

## Introduction

Emergency department (ED) crowding is a significant problem in many localities worldwide [1]. Usually, patients without a life-threatening condition are transferred to wards that offer ED support to decrease this overload [2]. However, these patients have a high risk of clinical deterioration, and often a limited number of electronic devices are available to monitor all these patients.

The Modified Early Warning Score (MEWS) is a physiologic scoring system for bedside assessment of medical-surgical patients to identify who are at increased risk of catastrophic deterioration [3,4]. Generally, higher MEWS scores indicate worsening clinical status. This score is usually applied to activate a rapid-response team (RRT), which is a healthcare team dedicated to early identification of and response to clinical deterioration in hospital-ward patients to avoid intensive care unit (ICU) admission or cardiac arrest.

The results of previous research about RRT implementation have ranged from a lack of evidence of benefit [5] to absolute decreases in in-hospital mortality and cardiopulmonary arrest of 0.06% and 0.15%, respectively [6].

This study aimed to evaluate whether the implementation of an RRT triggered by the MEWS ≥ 4 in high-risk wards offering ED support in a tertiary hospital is associated with decreased in-hospital mortality of these patients.

## Material and methods

A before-and-after cross-sectional study compared in-hospital mortality rates before (control period) and after (intervention period) implementing an RRT triggered by the MEWS≥ 4 in two tertiary wards that offer ED support. The Ethical Board of the Hospital das Clínicas of the Ribeirão Preto School of Medicine of the São Paulo University (process number 3.994.536) approved this research.

### Patients

This analysis included patients hospitalized in 51 hospital beds divided into two wards: 30 beds in the surgical ward and 21 beds in the internal medicine ward. These two wards are physically separated; the internal medicine ward is located on the second floor, and the surgical ward on the third floor of this hospital. The period from July 2015 through June 2017 (two years) was considered the phase before the implementation of this system to detect clinical deterioration (control period) and the period from July 2018 through July 2020 (two years) was considered the phase after this implementation (intervention period). From July 2017 through June 2018 was considered the time of simultaneous MEWS and RRT implementation in these two hospital wards.

We included patients age 18 or older with any medical diagnosis and hospitalized in one of the beds of these two wards without any electronic monitoring system and who stayed in the hospital for any length of time.

### Patient monitoring system

We evaluated the vital signs: heart rate, respiratory rate, blood pressure, temperature, and level of consciousness for all patients in these wards. Trained nurses manually evaluated these vital signs every six hours. The nurses entered these vital signs into the electronic medical record that automatically generates the MEWS. The last MEWS value for each patient was shown on a screen in front of the nursing station. The vital sign values were entered into the electronic medical record every six hours with a tolerance of one hour before or after this time. If the patient’s vital signs were not entered at the appropriate time, the system generated a visual alert on the same screen, and we considered the last observation inserted in the system for this analysis.

**Fig 1:**
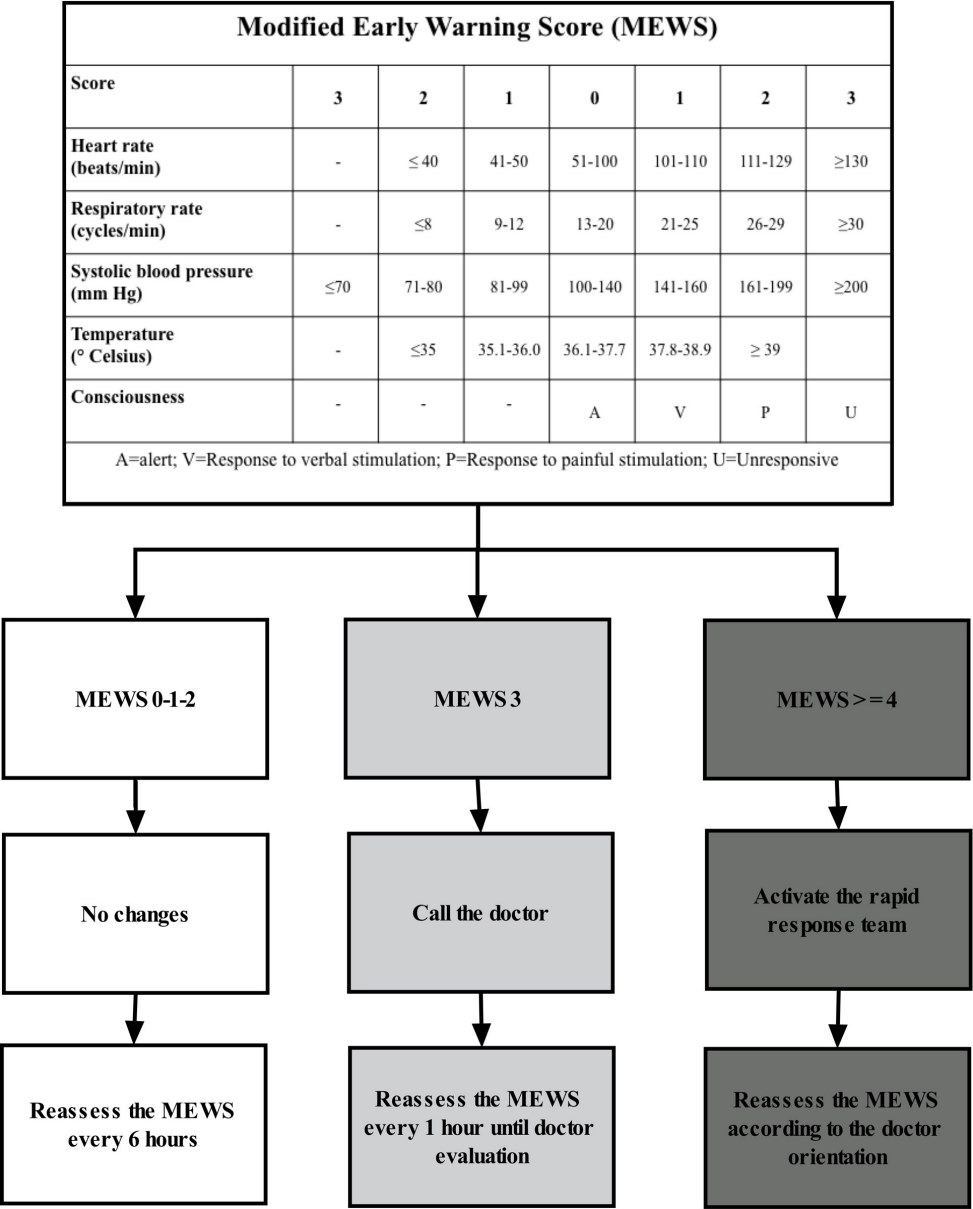
Type of response triggered by each Modified Early Warning Score (MEWS) value in the rapid-response team (RRT) implemented in the wards that offer emergency department support in this research.

We used four points in the MEWS as the cut-off to activate the RRT based on a previous pilot study performed in this same ward [7]. **Fig 1** shows how responses were triggered based on each MEWS value. When the MEWS achieved four points or more, the electronic system generated an alert on the screen in front of the nursing station. The nurse activated the RRT through one click on the screen. One doctor was exclusively dedicated to this task and one nurse from each ward was part of our RRT.

### Data collection

We obtained demographic and clinical data from patients’ medical records. The principal diagnosis that caused the patients’ hospitalization was classified according to the International Statistical Classification of Disease and Related Health Problems 10^th^ revision (ICD– 10/2019 Version) [8]. Due to the many different diagnoses observed, we decided to cluster the main diagnosis according to the hierarchical classification in the ICD-10 chapters. Just for this analysis, we divided all these diagnoses into two categories. The first category was called clinical diseases and it included all chapters, except for chapter XIX (Injury, poisoning, and certain other consequences of external causes) that constituted the second category renamed as external causes.

Once the RRT was activated, the doctors responsible for the clinical evaluation were oriented to fill out a specific electronic form about the patient’s condition instead of performing a traditional medical record register. In this electronic form, the doctors should specify if the patient exhibited a life-threatening condition. When this condition was observed, the doctors needed to detail the context (hypotension, sepsis, acute respiratory failure, altered consciousness, arrhythmia, acute heart failure, etc.). The primary outcome analyzed was the overall in-hospital mortality rate obtained through the electronic hospitalization system.

### Statistical analysis

We used the Shapiro-Wilk test to evaluate the type of distribution of the quantitative variables. The quantitative variables were expressed as mean and standard deviation (sd). Categorical variables were expressed in frequencies and percentages, and their association was analyzed through the chi-square test. Because of the large sample size, we used the standardized difference to compare the mean of the continuous variables and the prevalence of categorical variables between the two periods. The standardized difference was calculated using Cohen d. An absolute standardized difference of > 0.10 was considered to indicate an imbalance between the groups.

We calculated the prevalence ratio (PR) and its respective 95% confidence interval (95% CI) to evaluate the association of the two variables. The multivariable analysis employed a Poisson regression model with a robust variance estimator adjusted for after vs. before intervention, age, gender, type of ward, medical specialty, clustered main diagnosis, clinical diagnosis vs. external causes, and length of hospital stay. In a second step, the same Poisson regression model was performed for two clusters according to the type of ward (internal medicine or surgical). A two-tailed p-value < 0.05 was considered statistically significant. Statistical analysis and graphs were performed using the GraphPad Prism software version 7.00 (California, USA) and the STATA software version 13 (College Station, TX, USA).

## Results

**Fig 2:**
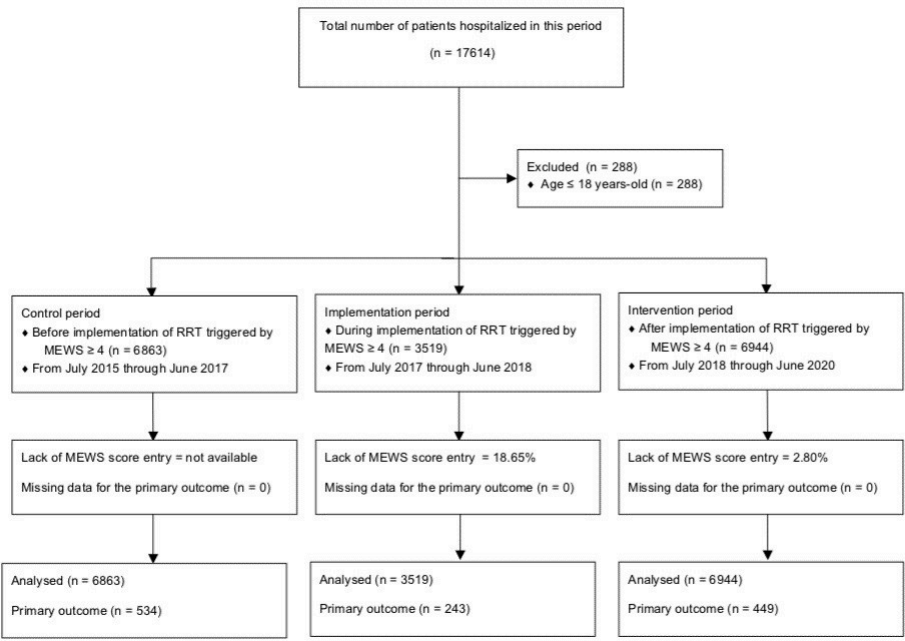
Flow diagram showing the patients included in this study.

We included 6863 patients hospitalized in one of these wards before implementation of the RRT (control period) and 6944 patients after implementation of this system (intervention period). Fig 2 is a flow diagram of the patients included in this study. Characteristics of the hospitalized patients before and after the intervention in these wards are presented in **Table 1**.

**Table 1:** Characteristics of the hospitalized patients before and after the implementation of the rapid-response team (RRT) triggered by MEWS≥4 in wards that offer emergency department support.

Characteristics		Before implementation N = 6863	After implementation N = 6944	Standardized Difference^1^
Demographic				
	Age; years, mean±sd	50.12±20.10	51.24±19.96	0.05
	Gender; male, n. (%)	3854(56.16)	4032(58.06)	0.04
Type of hospital ward, n. (%)				
	Internal Medicine	1812(26.40)	2062(29.69)	0.09
	Surgical	5051(73.60)	4882(70.31)	-0.09
Medical specialties, n. (%)				
	Internal Medicine	1812(26.40)	2062(29.69)	0.09
	Trauma Surgery	1742(25.38)	1872(26.96)	0.04
	Orthopedics	1455(21.20)	1446(20.82)	-0.01
	Gynecology	547(7.97)	407(5.86)	-0.18
	Head and neck surgery	416(6.06)	310(4.46)	-0.17
	Other	891(12.98)	847(12.20)	-0.03
Length of hospital stay, days; mean±sd		10.04±12.87	9.82±12.14	-0.01
Hospital bedtime, hours, mean±sd		121.39±155.16	119.52±139.03	-0.01
Clustered main diagnosis, n. (%)				
	Infectious and parasitic diseases	448(6.53)	426(6.13)	-0.03
	Neoplasms	990(14.43)	1115(16.63)	0.09
	Diseases of the blood and blood-forming organs	104(1.52)	127(1.83)	0.10
	Endocrine, nutritional, and metabolic diseases	208(3.03)	197(2.84)	-0.03
	Mental and behavioral disorders	92(1.34)	87(1.25)	-0.03
	Diseases of the nervous system	87(1.27)	97(1.40)	0.05
	Diseases of the eye, ear, and mastoid process	7(0.10)	6(0.09)	-0.09
	Diseases of the circulatory system	786(11.45)	733(10.56)	-0.05
	Diseases of the respiratory system	317(4.62)	292(4.21)	-0.05
	Diseases of the digestive system	1032(15.04)	1127(16.23)	0.04
	Diseases of the skin and subcutaneous tissue	144(1.66)	112(1.61)	-0.01
	Diseases of the musculoskeletal system and connective tissue	102(1.49)	137(1.97)	0.15
	Diseases of genitourinary system	351(5.11)	376(5.41)	0.03
	Pregnancy, childbirth, and the puerperium	172(2.51)	121(1.74)	-0.20
	Congenital malformations, deformations, and chromosomal abnormalities	22(0.32)	17(0.24)	-0.14
	Injury, poisoning, and certain other consequence of external causes	2031(29.59)	1934(27.85)	-0.04

sd–standard deviation;

^1^standardized difference was calculate using Cohen d; an absolute standardized difference of >0.10 was considered to indicate an imbalance between the groups.

**Fig 3:**
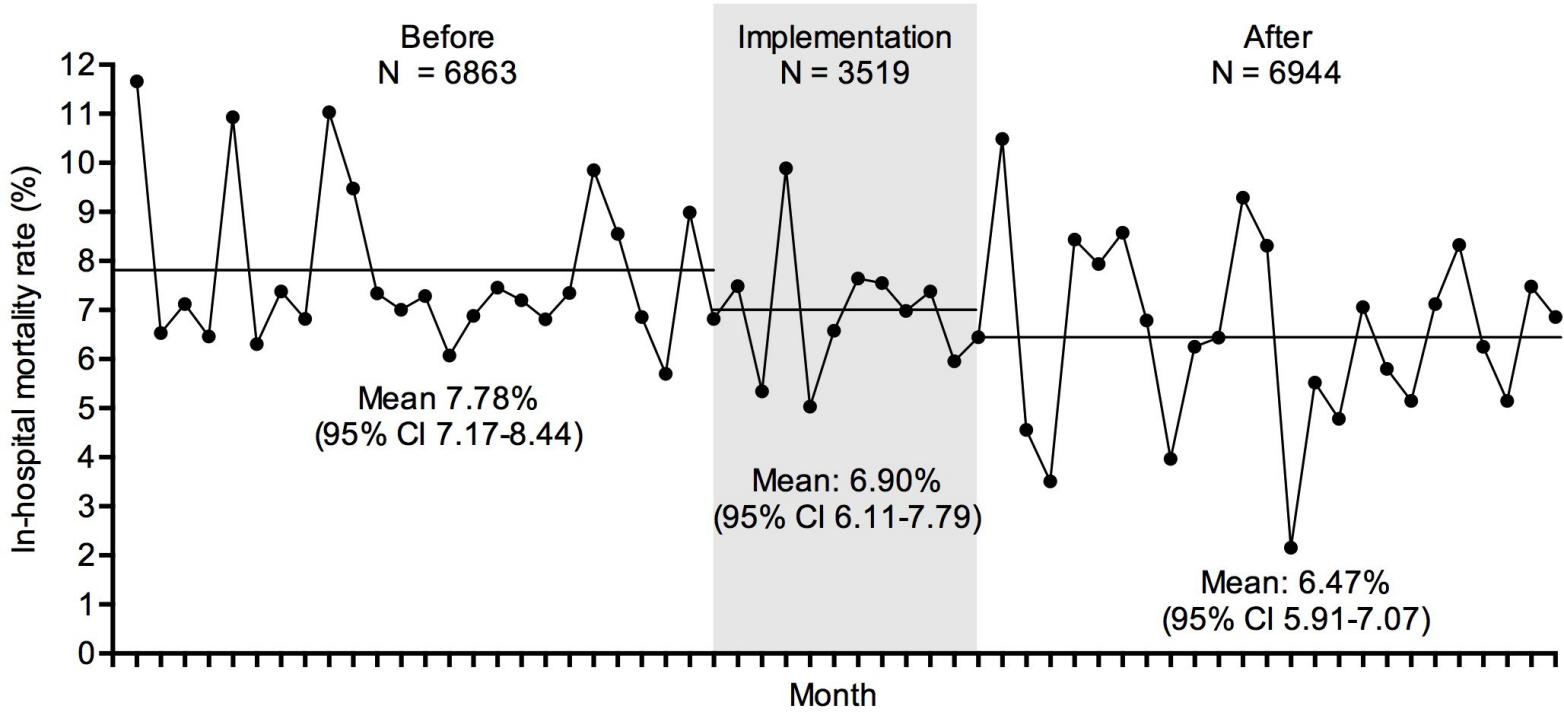
Panel data line graph illustrating the monthly in-hospital mortality rate in each study period according to the rapid-response team (RRT) trigged by the Modified Early Warning Score (MEWS) ≥ 4 implementation (before, during, and after). The horizontal line illustrated the mean of the in-hospital mortality in each period and these means and their respective 95% confidence interval (CI) were shown on the bottom of the panel.

We observed a statistically significant decrease in the in-hospital mortality rate after intervention compared to the control period, 449 deaths/6944 hospitalizations [6.47% (95% CI 5.91–7.07)] vs. 534 deaths/6863 hospitalizations [7.78% (95% CI 7.17–8.44)], p = 0.003 respectively; prevalence ratio (PR): 0.83, (95% CI 0.74–0.94), with an absolute risk reduction of -1.31% (95% CI -2.20 –-0.50) in the entire group. The analysis of the in-hospital mortality rate in the period during implementation of the RRT in these wards found an intermediate value between the control and intervention period, 243 deaths/3519 hospitalizations [6.90% (95% CI 6.11–7.79)]. **Fig 3** shows the monthly in-hospital mortality rate in these three periods.

Cluster analysis according to the type of ward (internal medicine and surgical) continued to exhibit a statistically significant decrease in the in-hospital mortality rate after intervention compared to the control period, 232 deaths/2062 hospitalizations [11.25% (95% CI 9.88–12.62)] vs. 260 deaths/1812 hospitalizations [14.35% (95% CI 12.73–15.96)], PR: 0.78 (95% CI 0.66–0.93); p = 0.004 for the internal medicine ward, and 217 deaths/4882 hospitalizations [4.44% (95% CI 3.90–5.00) vs. 274 deaths/5051 hospitalizations [5.42% (95% CI 4.80–6.05)] PR: 0.82 (95% CI 0.69–0.97), p = 0.025 for the surgical ward. Therefore, the internal medicine ward achieved a greater absolute risk reduction than the surgical ward (3.10% vs. 0.98%) **Table 2.**

**Table 2:** In-hospital mortality predictors in univariable and multivariable analysis.

	Univariable N = 13807			Multivariable^1^ N = 13807		
Parameter	PR	95% CI	*p*	PR	95% CI	*p*
After vs. before intervention	0.83	0.74–0.94	0.003	0.80	0.71–0.90	0.0001
Age	1.04	1.03–1.04	0.0001	1.04	1.03–1.04	0.0001
Gender (male vs. female)	1.04	0.92–1.18	0.481	–	–	–
Type of ward (internal medicine vs. surgical)	2.57	2.28–2.89	0.0001	2.69	2.15–3.39	0.0001
Medical specialty	0.99	0.95–1.03	0.589	–	–	–
Clustered main diagnosis	0.86	0.85–0.87	0.0001	0.89	0.88–0.91	0.0001
Clinical diagnosis vs. external causes	7.07	5.37–9.30	0.0001	1.07	0.76–1.51	0.674
Length of hospital stay	1.01	1.01–1.01	0.0001	1.02	1.01–1.02	0.0001

PR–prevalence ratio; CI–confidence interval;

^1^ adjusted for after vs. before intervention, age, gender, type of ward, medical specialty, clustered main diagnosis, clinical diagnosis vs. external causes, and length of hospital stay.

**Fig 4:**
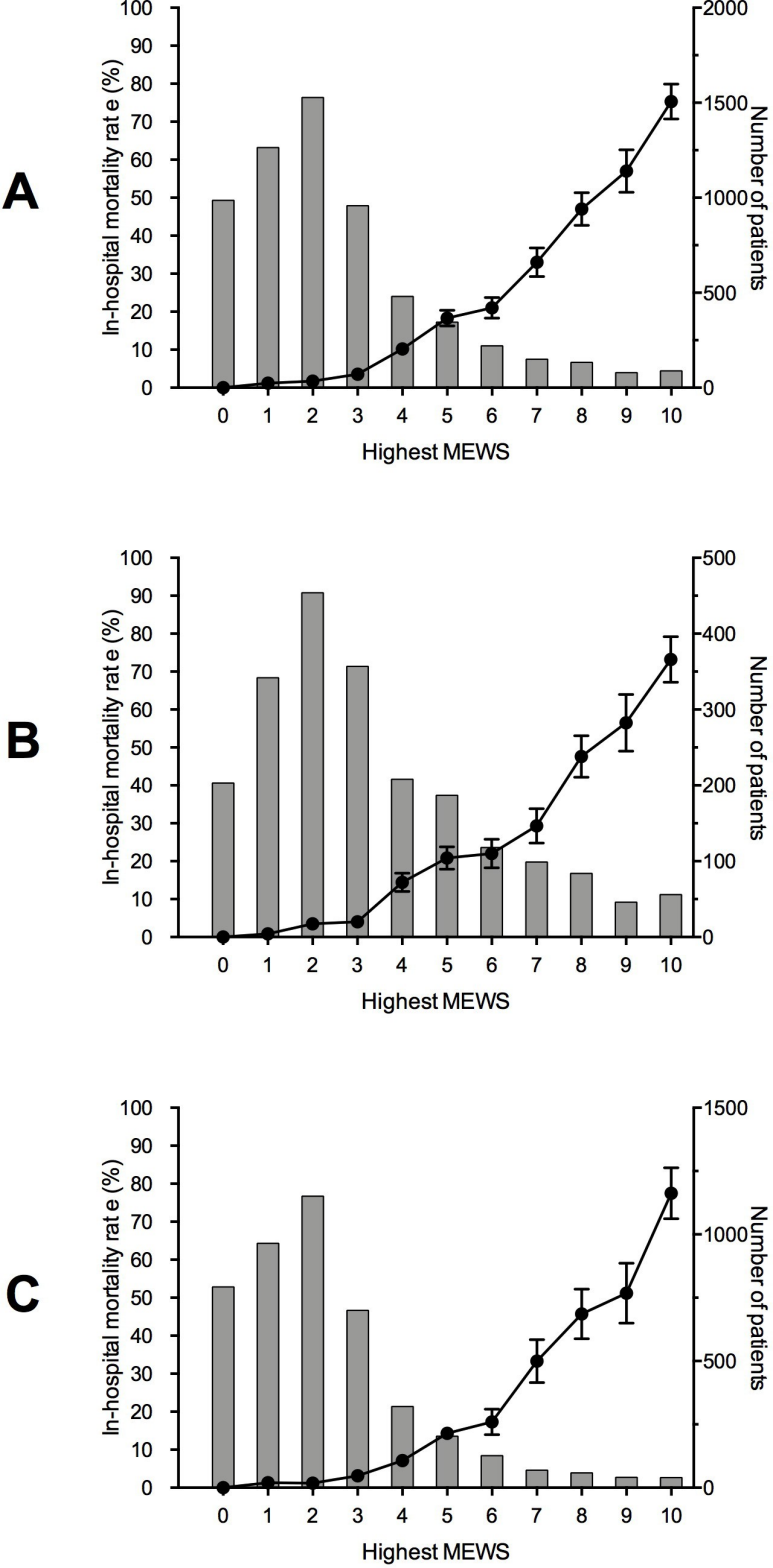
Line graph depicting the in-hospital mortality rate (%) in the left y-axis according to the highest Modified Early Warning Score (MEWS) registered during hospitalization. The small bars represent the standard errs. Bar graph showing the number of patients in the right y-axis for each highest MEWS value. A–All hospitalized patients; B–Only patients hospitalized in the internal medicine ward; C–Only patients hospitalized in the surgical ward.

The in-hospital mortality rate increased according to the rise in the highest MEWS observed during hospitalization. The in-hospital mortality rate increased sharply after four points in the MEWS in both wards **Fig 4.**

In multivariable analysis, the RRT triggered by the MEWS≥ 4 implementation remained associated with decreased in-hospital mortality rate after adjusting for confounding variables, PR: 0.80 (95% CI 0.71–0.90), p = 0.0001. Multivariable analysis using the same model was again divided into clusters according to the type of ward, and evidenced a decreased in-hospital mortality rate with RRT implementation in both types of wards, PR: 0.84 (95% CI 0.71–0.98), p = 0.034 for the internal medicine ward and PR: 0.78 (95% CI 0.66–0.93), p = 0.004 for the surgical ward **Table 2.**

There were 1749 system alert activations for patients whose MEWS achieved at least four points. The RRT doctors filled out 791 forms (45.23%) associated with these activations. Among the identified causes for RRT activations, 203/791 (25.66%) events were documented as a life-threatening condition. The main causes responsible for these life-threatening conditions are listed in **Table 3**. Identification of a life-threatening condition during the intervention period was associated with higher in-hospital mortality rate, PR: 2.46 (95% CI 1.96–3.07), p < 0.0001.

**Table 3:** Reasons for rapid-response team (RRT) activation.

Causes			N = 1749
Unidentified			958 (54.77)
Identified			791 (45.23)
	No life-threatening condition		588/791 (74.33)
	Life-threatening condition		203/791 (25.66)
		Hypotension	70/203 (34.48)
		Altered consciousness	58/203 (28.57)
		Sepsis	42/203 (20.69)
		Acute respiratory failure	17/203 (8.37)
		Other	13/203 (6.40)
		Arrhythmias	2/203 (1.00)
		Acute heart failure	1/203 (0.50)

## Discussion

This before-after study suggested a statistically significant association between RRT trigged by the MEWS≥4 implementation and a decrease in the in-hospital mortality rate in high-risk wards that offer ED support.

In a recent systematic review and meta-analysis, which included 30 different studies (before-after studies, cohort studies, and randomized-cluster trials), the RRT implementation was associated with a statistically significant decrease in the in-hospital mortality in 3,478,952 admissions; relative risk (RR): 0.88 (95% CI 0.83–0.93). A statistically significant reduction in cardiac arrest was also observed in 3,045,273 admissions; RR: 0.62 (95% CI 0.55–0.69). However, great heterogeneity was observed among these studies for both outcomes, I^2^ = 86%, and I^2^ = 71%, respectively [6].

In a nationwide implementation of an RRT trigged by the MEWS in 12 hospitals in the Netherlands (166,569 patients), a statistically significant decrease in the composite endpoint (cardiac arrest, unplanned ICU admission, or death per 1,000 admissions) was observed after RRT implementation compared to the previous phase; adjusted odds ratio (OR): 0.85 (95% CI 0.72–0.99), p = 0.036. The in-hospital mortality decreased from 20.4/1,000 admissions (95% CI 18.70–22.00) to 17.7/1,000 admissions (95% CI 16.20–19.20) after RRT implementation [9].

On the other hand, two cluster-randomized controlled trials failed to find a decrease in the in-hospital mortality after RRT implementation. In the MERIT study [10], the unexpected death rate was 1.18 per 1,000 admissions in the control group compared to 1.06 per 1,000 admissions in the RRT group, p = 0.564. In the study by Haegdorens et al. [11], the unexpected death was 1.50 per 1,000 admissions in the control group compared to 0.70 per 1,000 admissions in the intervention group, OR: 0.82 (95% CI 0.34–1.95). These two studies showed a similar low incidence of unexpected death, probably reflecting low-risk wards. To our knowledge, no related randomized clinical trial has been performed in exclusively high-risk wards.

We implemented an RRT trigger by the MEWS in a high-risk ward that offers ED support in a tertiary hospital. We found a high in-hospital mortality rate (65 per 1,000 admissions) in this setting as expected. Although we used the general in-hospital mortality rate instead of the unexpected death in our research.

Each study used a different trigger to activate the RRT. We opted to use the MEWS because it is a simple score calculated from the basic vital sign variables and was validated in a pilot study in these same wards [7]. Scientific evidence has showed that MEWS is an appropriate screening tool for major adverse events in hospitalized patients [3,12]. Van Galen et al. [13] found that patients with critical scores had statistically significant higher rates of unplanned ICU admissions (7.00% vs. 1.30%, p < 0.001), in-hospital mortality (6.00% vs. 0.80%, p < 0.001), and 30-day readmission rates (18.60% vs. 10.80%, p < 0.05). Our study observed a progressive increase in the in-hospital mortality according to the rise in the MEWS values.

Since 2007, the Royal College of Physicians [14] has recommended using the National Early Warning Score (NEWS) for routine clinical assessment of all adult patients in the hospital ward. NEWS showed an excellent accuracy to determine cardiac arrest, unanticipated ICU admission, and death through the area under the receiver-operating characteristic curve [15] of 0.72 (95% CI 0.69–0.76); 0.86 (95% CI 0.85–0.87) and 0.89 (95% CI 0.89–0.90), respectively [16]. The variables evaluated in this last score are similar to the MEWS, but NEWS also includes evaluation of the oxygen saturation and information about whether the patient was using supplemental oxygen. Despite this, research did not show a rise in the accuracy to determine the composite outcome (cardiac arrest, ICU transfer, mortality) with the inclusion of the oxygen saturation, MEWS, AUC: 0.75 (95% CI 0.74–0.76) vs. VitaPAC Early Warning Score (ViEWS), AUC: 0.75 (95% CI 0.74–0.76) vs. Standardized Early Warning Score (SEWS), AUC: 0.76 (95% CI 0.75–0.77) [17].

There is some controversy about the most appropriate MEWS cut-off point to activate the RRT [18]. Our investigation used a MEWS ≥ 4 points to activate this system according to a previous pilot study performed by our research group in the same ward [7]. In our opinion, the MEWS cut-off point should be individualized according to each ward profile.

Big data development allows the integrative use of information from the electronic medical record about the patient’s condition, such as laboratory test results, chronic coexisting conditions at admission, patient and hospital factors, and the vital signs. This integrative system can improve identification of patients in clinical deterioration and activate the RRT more precisely. Green et al. [19] demonstrated that the electronic cardiac arrest risk triage (eCART) exhibited better accuracy for predicting the composite outcome of in-hospital cardiac arrest, ICU transfer, and death within 24 h of observation, AUC: 0.81 (95% CI 0.79–0.80) compared to the NEWS, AUC: 0.71 (95% CI 0.71–0.72) and MEWS with an AUC of 0.69 (95% CI 0.69–0.70). A recent study found decreased mortality after implementing one of these systems in an intervention cohort compared to a comparison cohort, adjusted RR: 0.84 (95% CI 0.78–0.90), p < 0.001 [20]. However, these integrative systems are unaffordable for many hospitals due to the prohibitive cost of their implementation.

### Limitations

We highlight the major weakness of a before-after study is that observed changes attributed to the intervention could have been caused by temporal changes that occurred in these hospital wards over the studied timeline. Although there have been no critical changes in the infrastructure and human resources in these wards during this study, we cannot exclude that temporal trends could have interfered with these results. We analyzed the in-hospital mortality outcomes alone; and did not access other outcomes, such as cardiac arrest and unplanned ICU admission. However, we considered mortality to be the hardest endpoint. We used the general in-hospital mortality as the primary outcome instead of the unexpected death because to use this latter definition a careful review of each death is necessary. We did not exclude the do not resuscitate order (DNR) patients because our institution does not have a specific protocol for this condition. We cannot generalize these results for other ward profiles because we included only wards with high-risk patients in this research. The doctors filled out a low proportion (45.23%) of the forms about the reasons for the RRT activation; nevertheless, we think this did not interfere with the results. Usually, physicians did not fill out this specific form in situations where a life-threatening condition was not identified during clinical evaluation; however, the RRT doctors evaluated most of these cases. Because of the study design, other variables that affect the RRT activation and in-hospital mortality could not be considered in the multivariable analysis. Moreover, we cannot exclude that the reorganization of the vital sign monitoring system could have made an essential contribution to these findings. However, the RRT triggered by the MEWS implementation in these wards was an excellent opportunity to encourage the importance of vital sign assessment for all healthcare teams.

## Conclusion

Implementation of an RRT triggered by the MEWS≥ 4 in high-risk wards that offer ED support suggests an absolute risk reduction in the in-hospital mortality rate. Despite this, further cluster-randomized trials are needed to evaluate the real impact of this intervention in this setting.

## Supporting information

S1 Data (XLS)
